# Agonist Effects of Propranolol on Non-Tumor Human Breast Cells

**DOI:** 10.3390/cells9041036

**Published:** 2020-04-22

**Authors:** Lucía Gargiulo, Ezequiel Mariano Rivero, Nicolás di Siervi, Edgardo David Buzzi, Mariano Gabriel Buffone, Carlos Alberto Davio, Isabel Alicia Lüthy, Ariana Bruzzone

**Affiliations:** 1 Instituto de Biología y Medicina Experimental (IBYME-CONICET), Vuelta de Obligado 2490, C1428ADN CABA, Argentina; lupegargiulo@gmail.com (L.G.); ezequielmrivero@gmail.com (E.M.R.); mgbuffone@gmail.com (M.G.B.); isabel.luthy@gmail.com (I.A.L.); 2 Instituto de Investigaciones Farmacológicas (ININFA-UBA-CONICET), Facultad de Farmacia y Bioquímica, Universidad de Buenos Aires, Junín 956, C1113AAD CABA, Argentina; nicolasdisiervi@gmail.com (N.d.S.); carlosdavio@hotmail.com (C.A.D.); 3 Instituto de Investigaciones Bioquímicas de Bahía Blanca, CONICET-Universidad Nacional del Sur, Camino La Carrindanga km 7, 8000 Bahía Blanca, Argentina; ebuzzi@criba.edu.ar

**Keywords:** propranolol, β-blocker, biased agonism, MCF-10A, cell adhesion

## Abstract

The β-blocker propranolol (PROP) has been proposed as a repurposed treatment for breast cancer. The similarity of action between β-agonists and antagonists found on breast cells encouraged us to compare PROP and isoproterenol (ISO, agonist) signaling pathways on a human breast cell line. Cell proliferation was measured by cell counting and DNA-synthesis. Cell adhesion was measured counting the cells that remained adhered to the plastic after different treatments. Changes in actin cytoskeleton were observed by fluorescence staining and Western Blot. ISO and PROP caused a diminution of cell proliferation and an increase of cell adhesion, reverted by the pure β-antagonist ICI-118551. ISO and PROP induced a reorganization of actin cytoskeleton increasing F-actin, p-COFILIN and p-LIMK. While ISO elicited a marked enhancement of cAMP concentrations and an increase of vasodilator-stimulated phosphoprotein (VASP) and cAMP response element-binding protein (CREB) phosphorylation, PROP did not. Clathrin-mediated endocytosis inhibition or β-arrestin1 dominant-negative mutant abrogated PROP-induced cell adhesion and COFILIN phosphorylation. The fact that PROP has been proposed as an adjuvant drug for breast cancer makes it necessary to determine the specific action of PROP in breast models. These results provide an explanation for the discrepancies observed between experimental results and clinical evidence.

## 1. Introduction

Breast cancer is the third most common incident cancer overall considering both sexes. In 2018, 2,088,849 incident cases were estimated, the vast majority of them occurring in women [1]. For women, breast cancer is the leading cause of cancer death worldwide, with an estimated total of 626,679 deaths for this disease in 2018. These statistics demand either continuous research on new drugs or a repurposing of new uses of existing and well-characterized non-cancer drugs as treatments for cancer.

β-adrenergic receptors (β-AR), targets of endogenous catecholamines, are prototypical G protein-coupled receptors (GPCRs) that mediate the stress response. Preferentially β-AR couple to the heterotrimeric Gs protein activating adenylyl cyclase, increasing intracellular cyclic adenosine monophosphate (cAMP) levels and activating two major downstream effectors, protein kinase A (PKA) and the exchange protein directly activated by cAMP (EPAC). However, other mechanisms such as β-arrestin-dependent signaling [2], are well described. 

Although these receptors were originally associated with cardiovascular and central nervous system functions, in the last few years they have gained importance in the context of cancer, associating the β-adrenergic system with almost every hallmark of cancer [3,4]. Numerous research publications depicted the expression and role of β-AR on breast cancer [5,6,7]. Although still controversial, several reports, including ours, show that β-AR stimulation with specific agonists correlates with an inhibition of cell proliferation, cell migration and tumor growth [8,9,10,11]. In addition, on non-tumor breast models, β-AR stimulation increases the development and maturation of mammary epithelial structures [12] highlighting the importance of β-AR as a modulator of benign breast cell behavior.

Several publications describe the possible use of β-blockers (β-adrenergic antagonists) for the treatment of breast cancer [13,14,15]. Preclinical studies have suggested that β-adrenergic antagonists can inhibit multiple cellular processes, including cell proliferation and metastases [16]. However, the evidence regarding the effect of β-blockers on the breast is so far contradictory, showing inconsistencies related to the use of β-blockers and their specific role [17,18,19,20]. The similarity of action found between agonists and antagonists can be explained by the fact that several β-adrenergic antagonists do not really act as pure antagonists [21]. Some of them show partial agonist action, while others show an inverse agonist activity. It has been reported that carvedilol and PROP, both nonselective β-antagonists, stimulate ERK activation via a biased signaling mechanism in HEK 293S cells stably expressing human β2-AR [22]. Thus, β-adrenergic antagonists may activate numerous and different signaling pathways. 

Cell adhesion and proliferation are important processes involved in malignant cell progression. The aim of this study is to compare the effect of PROP with the agonist ISO on cell signaling pathways linked to these processes on breast cell lines. PROP behaves as an agonist on MCF-10A human breast cells. However, the signaling mechanism is different from the classical mechanism of agonists. 

## 2. Materials and Methods

### 2.1. Drugs, Reagents and Antibodies

(-)-Propranolol, (-)-isoproterenol, epidermal growth factor (EGF), hydrocortisone, bovine serum albumin (BSA), phenylarsine oxide (PAO) and poly-L-lysine, IV collagen and laminin were from Sigma-Aldrich (Saint Louis, MO, USA). Culture medium, fetal bovine serum (FBS) and other products for cell culture were from Invitrogen-Thermo Fisher Scientific (Waltham, MA, USA). Fibronectin was from Santa Cruz Biotechnology (Dallas, TX, USA). FuGENE^®^ was purchased from Promega (Madison, WI, USA). Human recombinant insulin was kindly donated by Denver Farma (Buenos Aires, Argentina). Methyl-[^3^H]-thymidine and [^3^H]-cAMP were purchased from PerkinElmer Life Sciences (Waltham, MA, USA). BMS-3 was from SynKinase and PF3758309 was from Medkoo Biosciences (Morrisville, NC, USA).

### 2.2. Cell Lines, Culture and Transfection

MCF-10A (ATCC^®^ CRL-10317™), MCF-7 (ATCC^®^ HTB-22™) and MDA-MB-231 (ATCC^®^ HTB-26™) cells lines were recently obtained from the American Type Culture Collection (ATCC, Manassas, VA, USA) and were cultured with HEPES-buffered DMEM:HamF12 culture medium (basal medium) supplemented with antibiotics (100 µg/mL streptomycin, 100 IU/mL penicillin), 10% FBS and 2 µg/mL human insulin. MCF-10A cells were also incubated with 20 ng/mL EGF and 0.1 µM hydrocortisone. Cells were sub-cultured by trypsinization (0.25% trypsin–0.025% EDTA) and the medium was changed three times a week. For transfection, 1.5 × 10^5^ cells/well were seeded into 12 well-plates in complete medium. Cells were transfected with the empty vector (mock) or β-arrestin1 dominant negative mutant [23] as previously described [10].

### 2.3. Cell Proliferation

Cells were seeded in 12-well plates (15 × 10^3^ cells/well) for cell-counting or 96-well plates (5 × 10^3^ cells/well) for DNA synthesis experiments and grown during 24 h in complete medium. After 24 h the medium was changed to 2% charcoal-stripped FBS and the cells were treated with the compounds described. The treatment was repeated an additional day (with the addition of 0.2 μCi methyl-[^3^H]-thymidine/well for DNA synthesis experiments). Proliferation response was evaluated harvesting cells in Cell Harvester 8 (Nunc, Roskilde, Denmark) and counting in a liquid scintillation counter at 48 h or by cell-counting using Neubauer chamber. The results are expressed as percentage of the control incubated in the absence of any compound.

### 2.4. Resistance to Trypsinization

Cell adhesion was measured as previously described [24]. Briefly, the medium was removed and cells were treated with or without the test compounds in basal medium for 15 min. The medium was subsequently removed and cells were incubated in Mg^2+^/Ca^2+^-free PBS containing 0.5 mM EDTA and 0.25% trypsin with constant agitation at room temperature during 15 min. Cells that resisted the treatment and remained adhered to the plastic were harvested following additional 30 min incubation in Mg^2+^ and Ca^2+^-free PBS containing 2.5 mM EDTA and 1.25% trypsin and were counted as the number of attached cells. The percentage of adherent cells was calculated as follows: attached cells × 100/(attached cells + detached cells).

### 2.5. Fluorescence Staining

Cells were seeded and cultured for 24 h in 6-well plates containing glass coverslips pre-coated with poly-L-lysine, and then grown in basal medium for 18 h. Coated experiments were performed coating coverslips overnight with 2 μg/mL fibronectin, 20 μg/mL laminin (at room temperature) or 50 μg/mL type IV collagen (at 4 °C). They were then blocked 40 min at 37 °C with 1% BSA. Fibronectin micropattern essay was performed using CYTOOchip following manufacturer’s instruction (CYTOO, Grenoble, France). On the experiment day, cells were treated or not treated for 5 min with the indicated compounds and then fixed with 4% paraformaldehyde in PBS for 10 min, permeabilized 15 min in blocking buffer (PBS, 0.3% Triton X-100, 0.2% BSA) and incubated 30 min with phalloidin–tetramethylrhodamine B isothiocyanate (1:1000, DAKO-Agilent, Santa Clara, CA, USA) or Alexa fluor™ 488 phalloidin (1:100, Molecular Probes-Thermo Fisher Scientifics). For CYTOO experiments, cell nuclei were stained with 4´,6-diamidino-2-phenylindole 1:500 (Sigma-Aldrich) for 10 min.

### 2.6. Western Blot

Cells were seeded into 6-well plates (2.5 × 10^6^ cells/well) and cultured for 24 h in complete medium followed by 24 h in basal medium. Cells were subsequently incubated with the indicated compounds, rapidly washed with ice-cold PBS and lysed in RIPA buffer (10 mM Tris, pH = 7.5, 150 mM NaCl, 2 mM Na ortho-vanadate, 0.1% SDS, 1% Igepal, 1% Na deoxycholate). Equal amounts of protein were separated on SDS-PAGE and transferred to nitrocellulose (Millipore, Billerica, MA, USA). Separation of fibrillar form of actin (F-actin) and globular actin (G-actin) was performed as previous described [25]. Briefly, cells were extracted using extraction buffer (50 mM Tris, 150 mM NaCl, 1% Triton X-100, 0.1% SDS and protease and metalloprotease inhibitors, pH 7.8) and vortexed vigorously. The sample was subsequently centrifuged at 13,000 rpm for 5 min at 4 °C. The Triton-insoluble content of the protein extract was obtained by removing the supernatant fluid (triton-soluble content, G-actin). Triton-insoluble fraction (F-actin) was resuspended in sample buffer and after centrifugation for 5 min at 13,000 rpm, the supernatant was obtained. A total of 5% β-mercaptoethanol and 0.0005% bromophenol blue were added to the supernatant. The extracted proteins were separated by SDS-PAGE and immunoblotted. Antibodies against p-COFILIN (Cat# 3311, RRID:AB_330238), COFILIN (Cat# 3318, RRID:AB_2080595), p-LIMK (Cat# 3841, RRID:AB_2136943), p-VASP(Ser157) (Cat# 84519, RRID:AB_2800039) y p-CREB (9198, RRID:AB_2561044) were from Cell Signaling Technology (Danvers, MA, USA) and were used at a 1:500 concentration. Extracellular-signal-regulated kinase 1 (ERK1) (sc-94, RRID:AB_2140110), ERK2 (sc-154, RRID:AB_2141292) and ACTIN (sc-58673, RRID:AB_2223345) were from Santa Cruz Biotechnology (used at 1:100). Immunoblots were revealed by chemiluminescence using horseradish peroxidase–conjugated secondary antibody (Amersham Biosciences GE Healthcare, Marlborough, MA, USA). Immunblots for ERK1 or ERK2 were used for protein loading normalization. Chemiluminescence detection was performed using an enhanced detection solution (1.25 mM luminol, 0.2 mM p-coumaric acid, 0.06% (v/v) hydrogen peroxide, 100 mM Tris-HCl pH 8.8). Immunoblots were exposed to autoradiographic film (Thermo Fisher Scientific, Waltham, MA, USA) and quantified by densitometry with ImageJ 1.46r (US National Institute of Health).

### 2.7. cAMP Quantification

cAMP content was quantified using a competitive radio-binding assay for PKA using [^3^H]-cAMP [26]. Cells were seeded in 24-well plates (1.7 × 10^5^ cells/well) in complete medium. After 24 h, the medium was removed and cells were incubated in RPMI medium without phenol red at 37 °C with ISO or PROP in the presence of 1 mM 3-isobutyl-methylxantine at 37 °C. Ethanol was added to stop the reaction. The extracts were centrifuged for 3 min at 3000× *g* and the recovered supernatant was evaporated and then resuspended in 50 mM Tris-HCl, pH 7.4, 0.1% BSA for cAMP quantification. The data shown are the result of duplicates from at least three independent experiments.

### 2.8. Data and Statistical Analysis

Experiments were repeated at least three times with similar results. Graph Pad Prism V.5 was used to perform statistical analysis as Student´s t-test, ANOVA) or Kruskal–Wallis followed by the corresponding post-test. A value of *p* < 0.05 was defined as threshold. Differences were considered significant when *p* < 0.05.

## 3. Results

### 3.1. Comparison of ISO and PROP Effect on Cell Proliferation and Cell Adhesion

**Figure 1:**
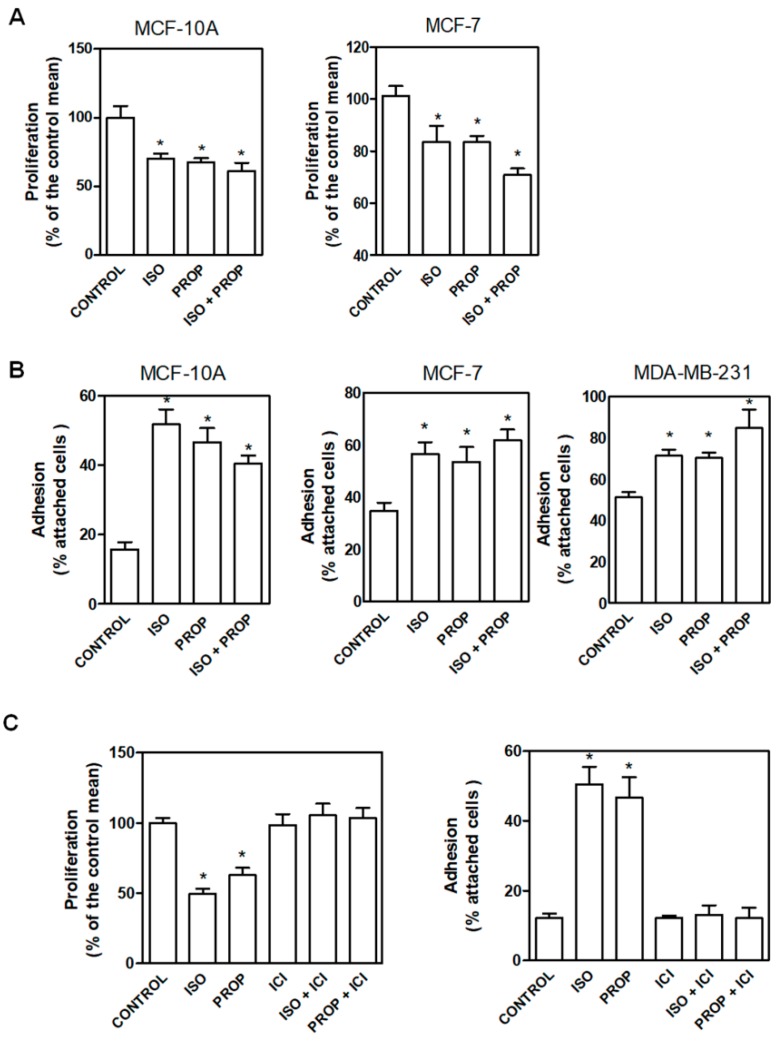
Effect of isoproterenol (ISO, 1 μM)) and propranolol (PROP, 1 μM) on cell proliferation and cell adhesion of tumor and non-tumor breast cells. (**A**) Cells were incubated with ISO, PROP or both and cell proliferation was measured in MCF-10A and MCF-7 cells. (**B**) Effect of ISO and/or PROP on cell adhesion of MCF-10, MCF-7 and MDA-MB-231 cells. (**C**) Effect of a β2-AR selective antagonist ICI-118551 (ICI, 10 μM) on ISO or PROP effect on MCF-10A cell proliferation or cell adhesion. ICI was pre-incubated 20 min before ISO or PROP treatment. Statistical significance was assessed using ANOVA and Bonferroni’s test or Kruskall–Wallis–Dunn’s Multiple Comparison Test. * *p* < 0.05. Data are representative of three independent experiments.

In order to compare the effect of the classic β-adrenergic agonist ISO with that of the antagonist PROP, cells were incubated with these compounds (1 μM) and cell proliferation and adhesion were analyzed (Figure 1). We previously described that PROP produces growth inhibition in MDA-MB-231 cell line growing in vivo. Here, we observed that ISO and PROP caused a significant decrease in in vitro cell proliferation of MCF-7 and MCF-10A cells (Figure 1A). In addition, both compounds increased cell adhesion in MCF10-A, MCF-7 and MDA-MB-231 cells (Figure 1B). We also previously reported that PROP behaves, in some breast cancer experimental models, as a partial antagonist only when the agonist is present [11]. The effect of the incubation with both ISO and PROP on cell adhesion and proliferation in all the cell lines analyzed was the same as that produced by each of them separately (Figure 1). Tumor cells were included in order to assess if PROP also behaved as agonist in these cells. To further describe this PROP effect, MCF-10A cells were incubated with ICI-118551 (ICI, a β2-AR pure selective antagonist). ICI was able to reverse the agonist effect and PROP effect, suggesting an agonist action of PROP via the β2-AR subtype.

### 3.2. Actin Reorganization Induced by ISO and PROP

**Figure 2:**
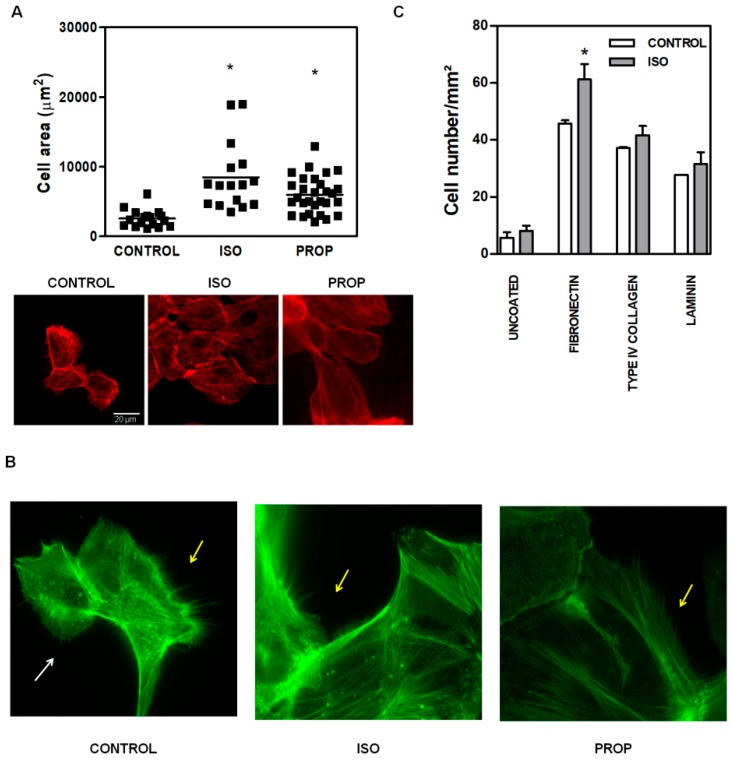
Increase of cell area and changes in actin cytoskeleton of MCF-10A cells induced by isoproterenol (ISO) and propranolol (PROP). (**A**). Fluorescence staining with phalloidin (red). Cells were treated or not treated (CONTROL) during 10 min with 1 μM ISO or 1 μM PROP and adherent cell area was quantified automatically with ImageJ. The scale is the same for every photograph. (**B**) Fluorescence staining with green phalloidin. White arrows show lamellipodia, while yellow arrows depict filopodia. (**C**) Agonist adhesion to specific extracellular matrix protein fibronectin, type IV collagen, laminin compared with uncoated control. Statistical significance was assessed using Kruskal–Wallis test (**A**) or ANOVA Bonferroni test (**C**). * *p* < 0.05. Data are representative of three independent experiments.

Given the multitude of pathways triggered after β-AR activation, we focused on the molecular signaling pathways involved in actin cytoskeleton reorganization in non-tumor cells, as it is linked to cell adhesion. ISO and PROP augmented the size of the attached cell area (Figure 2A, the scale is the same for every photograph). The incubation with both ISO and PROP quickly reorganized actin cytoskeleton. An evident and significant reduction of the number of filopodia and lamellipodia was observed after ISO and PROP treatment (68% and 82% of reduction respectively compared to control, Figure 2B). To study the specific extracellular matrix protein to which the agonist adheres, adhesion essays over glasses coated with different matrices were performed. While all matrices, fibronectin, type IV collagen and laminin induced an increase in cell adhesion and adhered cell area (compared to the uncoated control), the agonist induced adhesion specifically to fibronectin (Figure 2C).

**Figure 3:**
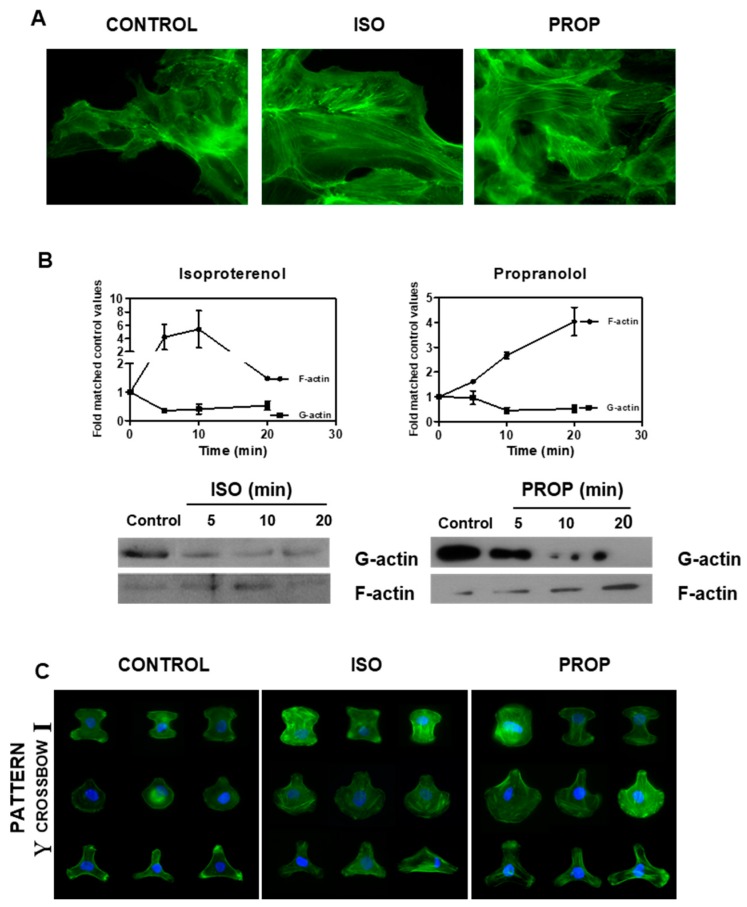
Isoproterenol (ISO) and propranolol (PROP) induce F-actin stabilization on MCF-10A. (**A**) Fluorescence staining with green phalloidin after 10 min of treatment with ISO or PROP. (**B**) Western Blot for F-actin and G-actin after ISO or PROP treatment. (**C**) Actin reorganization on a single cell is shown by adhesion to specific Y, crossbow or I fibronectin micropattern. Data are representative of three independent experiments.

ISO as well as PROP increased the F-actin observed by staining with phalloidin and by Western Blot (Figure 3A,B). The fibrillar disposition of actin in a single cell was easily observed using CYTOO chips, where cells adhere on a defined “Y”, “crossbow” or “I” fibronectin pattern (Figure 3C).

### 3.3. Signal Pathway Regulating Cell Adhesion Induced by ISO or PROP

**Figure 4:**
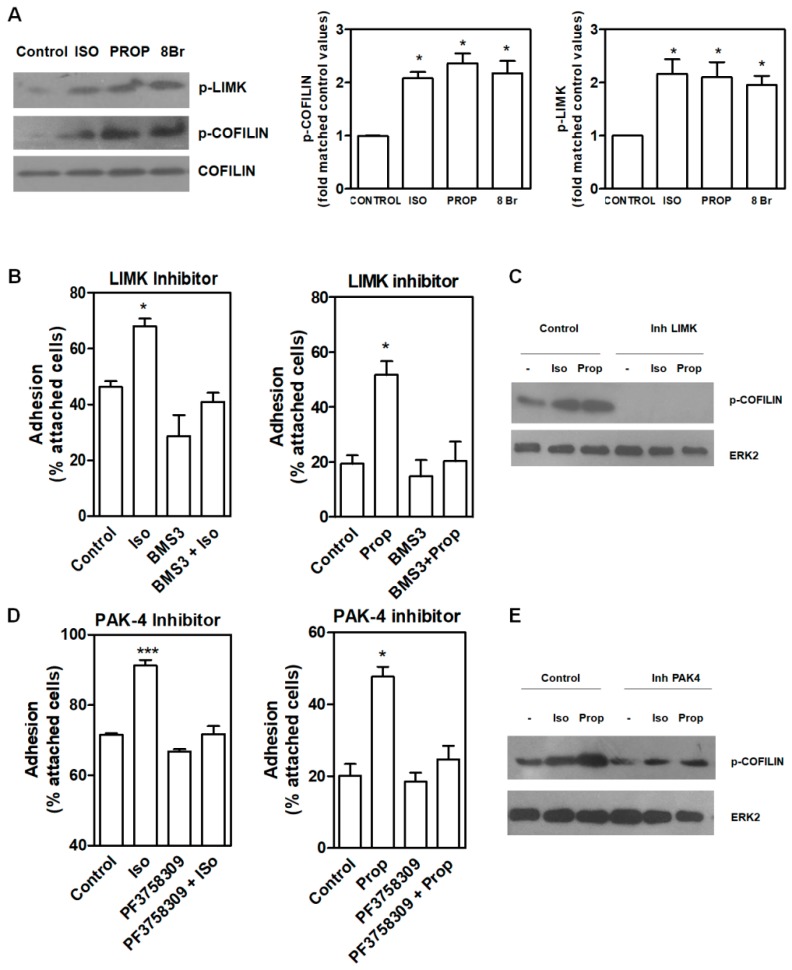
LIMK and COFILIN mediate isoproterenol (ISO) and propranolol (PROP) induced cell adhesion in MCF-10A cells. (**A**) Western Blot of p-LIMK and p-COFILIN after ISO, PROP or the cAMP analog 8Br-cAMP (8Br) treatment. (**B**) Effect of LIMK inhibitor (20 μM BMS-3) on cell adhesion induced by ISO or PROP. (**C**) Western Blot of p-COFILIN after BMS-3 treatment. ERK2 was used as loading control. (**D**) Effect of a PAK-4 inhibitor (10 μM PF3758309) on cell adhesion induced by ISO or PROP. (**E**) Western Blot of p-COFILIN after PAK-4 inhibitor treatment. ERK2 was used as loading control. Statistical significance was assessed using ANOVA Dunnett test. * *p* < 0.05, *** *p* < 0.001. Data are representative of three independent experiments.

COFILIN is a member of a family of essential conserved small actin-binding proteins involved in assembly and disassembly of actin filaments. COFILIN activity is regulated, among other mechanisms, by phosphorylation at Ser-3 caused by the kinase LIMK. This phosphorylation inhibits actin binding, severing, and depolymerizing F-actin. Our results showed that after 10 min of incubation with ISO and PROP, COFILIN was phosphorylated on Ser3, as well as LIM Kinase 1 (LIMK) at Thr508 (Figure 4A). In addition, incubation with 8-Br-cAMP (8Br), a membrane-permeable derivative of cAMP, also induced the phosphorylation of these proteins. In the presence of a specific LIMK inhibitor (20 μM BMS-3), cell adhesion induced by ISO or PROP was abolished (Figure 4B), thus suggesting that p-COFILIN is a mediator of both ISO and PROP-induced cell adhesion. To reinforce this, PAK-4 inhibitor (10 μM PF3758309) was used. It was described that PAK4, as well as PAK1, phosphorylate LIMK1 at Thr508 [27]. In addition, PAK4 phosphorylates and inactivates the slingshot homolog 1 (SSH1) phosphatase, increasing p-COFILIN and leading to a stabilization of the actin filaments [28]. Therefore, inhibition of PAK-4 also abolished ISO and PROP-induced cell adhesion and reduced COFILIN phosphorylation (Figure 4D–E).

**Figure 5:**
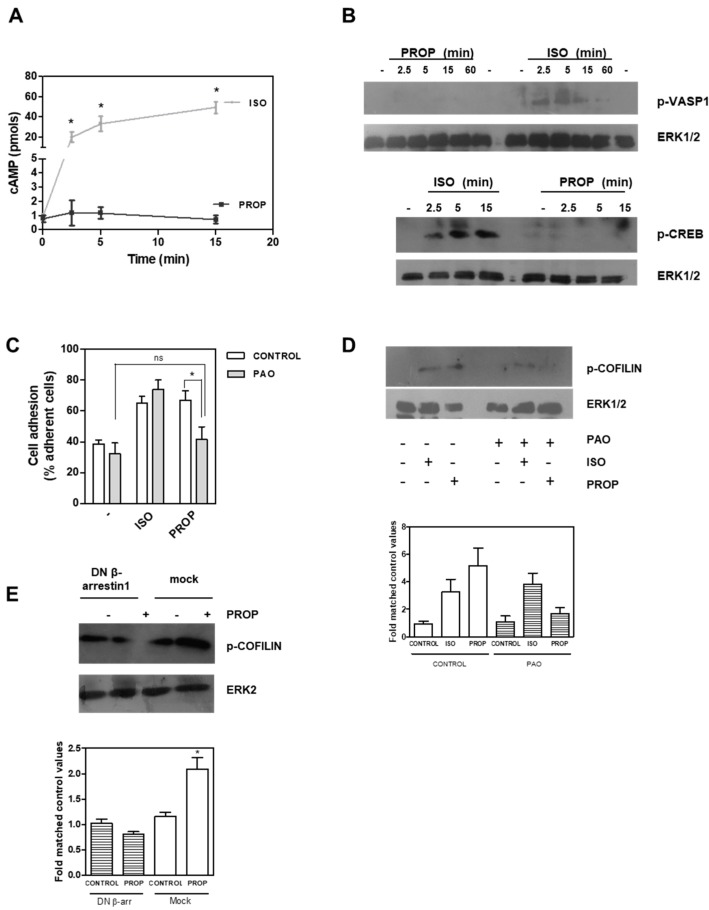
(**A**) Time course of intracellular cAMP production in MCF-10A cells in the presence of 3-isobutyl-methylxantine after isoproterenol (ISO) or propranolol (PROP) incubation. (**B**) ISO but not PROP induces a time dependent phosphorylation of cAMP response element-binding protein (CREB) and vasodilator-stimulated phosphoprotein (VASP) proteins measured by Western Blot. (**C**) Effect of phenylarsine oxide (PAO) on ISO and PROP induced-cell adhesion. (**D**) Western Blot of p-COFILIN after ISO and PROP treatment in the presence (+) or absence (−) of PAO. (**E**) Western Blot of p-COFILIN after PROP treatment in cells transfected with β-arrestin 1 dominant–negative (DN) or with the empty vector (mock). ERK1/2 or ERK2 were used as loading controls. Statistical significance was assessed using ANOVA Bonferroni test. * *p* < 0.05, n.s: non-significant difference. Experiments were repeated three times with similar results.

It is well known that β-AR stimulation causes an increase in cAMP levels, and our results showed an agonist effect of PROP. We therefore quantified cAMP levels after 1 μM ISO or PROP treatment. ISO elicited a marked enhancement of cAMP concentrations while PROP did not change cAMP levels, even in the presence of the phosphodiesterase inhibitor, 3-isobutyl-methylxantine (Figure 5A). VASP belongs to the Ena/VASP family of adaptor proteins linking the cytoskeletal system to signal transduction pathways and it functions in cytoskeletal organization. ISO but not PROP was also able to phosphorylate VASP in Ser157, the major PKA phosphorylation site of VASP, thus suggesting that ISO regulates the cytoskeleton, at least in part, via the classical pathway involving cAMP, while PROP does not (Figure 5B). In addition, ISO but not PROP induced a time-dependent phosphorylation of CREB (Figure 5B). Furthermore, whereas PAO, a membrane-permeable protein-tyrosine phosphatase inhibitor that blocks clathrin-mediated endocytosis, inhibited both PROP-induced cell adhesion and PROP-induced COFILIN phosphorylation, it did not inhibit ISO-induced effects on the same parameters (Figure 5C,D). Interestingly, using a β-arrestin1 dominant–negative (DN) mutant, which disrupts receptor internalization in intact cells, COFILIN phosphorylation induced by PROP was abrogated (Figure 5E).

All together, these results strongly suggest that the β-blocker propranolol is a biased agonist that regulates cell adhesion in non-tumor MCF-10A human breast cells. Evidence suggests a mechanism involving β1-arrestin as mediator of clathrin-dependent endocytosis. The results described herein allow us to partly explain the discrepancies observed between experimental results and clinical evidence.

## 4. Discussion

In the era of drug repositioning, the new uses of existing drugs in oncology are interesting and they encourage new strategies for cancer treatment because of their proven security and low costs. PROP has been described as a nonselective beta-blocker inhibiting sympathetic effects acting through β-AR. Clinical uses of PROP range from treatment of cardiovascular diseases to management of migraine, essential tremors, anxiety, portal hypertension, hyperthyroidism, pheochromocytoma and infantile hemangioma as reviewed in [29]. During the last few years, several studies suggested a role of PROP in the treatment for cancer, although its working mechanism is unknown [14,15,16].

In relation to breast cancer, retrospective studies reported improved survival with reduction in the risk of recurrence in women receiving this β-blocker for their hypertension [18,20]. On the other hand, other studies conclude that the use of β-blockers has no effect on the prognosis or survival of patients with breast cancer [17,30]. Furthermore, other retrospective studies associated the use of β-blockers with an increased risk of breast cancer and recurrence rates [19]. All these works demonstrate inconsistencies related to the use of β-blockers and their specific role in the breast. The global analysis of these studies thus requires taking into account the types of tumors analyzed, the β-blockers used, doses, duration and the time of administration, as well as the specific action of these compounds on the β-RA.

Previous work claims that numerous β-adrenergic antagonists relevant for their clinical use do not really act as pure antagonists [21]. Some of them act as partial agonists, while others show inverse agonism. Moreover, compounds originally defined as antagonists have been reclassified as biased agonists. The latter are ligands with the potential to generate different efficiencies for different responses of the same receptor [31]. Biased signaling strongly suggests the existence of different conformations of the receptor, preferentially stabilized by different ligands, which lead to the activation of different signaling pathways [32]. The nonselective β-antagonist carvedilol is a biased agonist, signaling independently on Gαs and dependently on β-arrestin [33]. In fact, many adrenergic antagonists reclassified as biased agonists have a bias for β-arrestins [34,35]. In line with this, it has been reported that in hippocampal neurons, PROP acts as an agonist, with a similar final action to that of ISO but through a different mechanism. This compound inhibited G-mediated signaling, activating the β-arrestin, and ERK1/2 pathways [36]. Additionally, in a model of antinociception in rats, PROP displayed mixed agonist and antagonist actions on the β2-AR as well as on the other β-AR isoforms [37]. Therefore, the β-adrenergic antagonists used in the clinic may be activators of numerous and different signaling pathways. Given that the physiological consequences and their implications are currently unknown, research focus on these mechanisms has clinical relevance because of the aforementioned proposal to use PROP as adjuvant therapy in breast cancer.

The ISO effect on in vitro parameters associated with breast tumor progression has been previously studied [6,10,11,38]. However, the study of PROP effect on in vitro parameters in the literature is controversial and scarce. Here, we found an agonist action of this classical β-adrenergic antagonist both in cell proliferation and adhesion, at a dose of 1 μM via the β2-AR. Most of the experiments were completed at both concentrations (0.1 and 1 μM), giving similar results. Pasquier et al. reported a decrease in the proliferation of tumor mammary cells using PROP concentrations between 10–50 μM [39]. A decrease in viability of the human breast cancer triple negative MDA-MB-231 cells by PROP was also described at 100 and 200 μM concentrations, arresting cell cycle progression at G0/G1 and S phase and inducing cell apoptosis by inhibiting the activation of ERK1/2 and the expression of cyclo-oxygenase 2 [40]. Nevertheless, non-specificity was observed at such high concentrations of PROP. It was reported that, in fact, at high doses, PROP can inhibit either phospholipase D or protein kinase C, due to its interactive membrane properties. PROP can act as an amphipathic cationic drug, penetrating the membrane, binding to specific phospholipids and redirecting neutral phospholipid biosynthesis to acidic phospholipids [41,42]. This inhibition was observed at concentrations 100 times higher than that necessary to antagonize the receptor, suggesting that the effect of PROP at high doses is not mediated by β-AR but by its membrane stabilizer properties [42]. These PROP effects, independent of β-AR, increase the level of complexity for the use of PROP in signal transduction studies. We already described PROP actions with lower concentrations taking into account unspecific actions at higher concentrations. Moreover, the peak plasma levels of PROP following usual clinical doses range from 20 to 200 ng/mL (roughly 0.1 µM-1 µM) [43]. We then considered the use of 1 μM concentration of PROP appropriate in this work to minimize non-specific effects, and therefore attribute the effects only to the β-adrenergic receptor. In addition, the pure antagonist ICI was able to reverse the effect of both ISO and PROP on cell adhesion and proliferation. ICI has a greater affinity for β2-AR than for β1 and β3-AR [44] and β2 is the main β subtype expressed in MCF-10A cells [24], thus corroborating PROP agonist action in β2-RA in these cells. In addition, ISO and PROP doses were compatible with those operating through the receptor. According to this, it was described that ICI blocked the effects of ISO and PROP in other models [22,45]. In β2-RA-overexpressing HEK-293 cells, PROP and other nonselective β-adrenergic antagonists showed the same effect that either ISO or SALB had on ERK1/2 signaling but not over other pathways normally activated by these compounds [22,33].

In the present investigation, we focused on signaling pathways involved in cell adhesion in non-tumor cells. We previously reported β2-RA as a regulator of cell adhesion [24]. Actin cytoskeleton architecture and its reorganization has been associated with numerous cellular processes, including cell adhesion, migration and apoptosis. F-actin network is a dynamic process controlled by the action of different actin binding proteins. Stabilization of F-actin filaments has been associated with the inhibition of migration and invasion in lung tumor cells through the regulation of stress fibers and the maturation of focal adhesions [46]. According to this, diminution of cell migration and increase in cell adhesion previously reported in MCF-10A [10,12] could be mediated by the stabilization of the F-activity observed here.

The COFILIN family (actin-depolymerizing factor (ADF)/cofilin) regulates the actin network by increasing the off-rate of actin monomers from the pointed end of actin filaments and filament severing. Phosphorylation of serine 3 (Ser3) by the LIMK results in COFILIN inactivation, whereas dephosphorylation by SSH phosphatases results in their reactivation. The increase in COFILIN phosphorylation by LIMK and thus its inactivation could both explain the increase of F-actin found in the present work by both ISO and PROP. In a completely different model, such as the airway smooth muscle, ISO decreased COFILIN phosphorylation in a PKA-dependent manner [47]. The increase found in the present investigation could be due in part to PKA in the case of ISO but by a different mechanism with PROP because no stimulation of cAMP and p-CREB was found with this compound. In primary lymphocytes and cultured cells, the scaffolding of COFILIN by β-arrestins was described [48]. In this regard, a DN of β-arrestin1 abrogates COFILIN phosphorylation induced by PROP. The inhibition of the action by the internalization inhibitor PAO could also suggest a mechanism mediated by receptor internalization, which also points to arrestins.

Using pharmacological inhibitors, we demonstrated that ISO and PROP-induced adhesion in MCF-10A cells is dependent on PAK, LIMK and COFILIN. The mechanisms upstream of LIMK and how these pathways are related to members of the GTPase family remain to be elucidated. Phosphorylation of VASP at the Ser157 residue reduces its association with actin having a negative effect on actin polymerization [49]. We observed an increase in VASP phosphorylation after ISO and 8Br, a permeable analog of cAMP and activator of PKA. A similar increase in VASP phosphorylation was reported in airway smooth muscle by β2-adrenergic agonists in a PKA-dependent manner [50] as well as and in cardiac myocytes [51].

## 5. Conclusions

In conclusion, the non-selective antagonist PROP behaves in the non-tumor line MCF-10A as an agonist, increasing adhesion and decreasing proliferation, via the β2-RA. In this work, it was possible to demonstrate that the adhesion induced by ISO and PROP is mediated by PAK4/LIMK/COFILIN proteins, and to suggest that whereas ISO involves a cAMP pathway, PROP acts through an internalization pathway involving arrestins and possibly clathrin internalization.
